# Natriuretic Peptides as a Prognostic Marker for Delirium in Cardiac Surgery—A Pilot Study

**DOI:** 10.3390/medicina56060258

**Published:** 2020-05-27

**Authors:** Thomas Saller, Sven Peterss, Patrick Scheiermann, Daniela Eser-Valeri, Johannes Ehler, Dirk Bruegger, Daniel Chappell, Othmar Kofler, Christian Hagl, Klaus Hofmann-Kiefer

**Affiliations:** 1 Department of Anaesthesiology, University Hospital, LMU Munich, 81377 Munich, Germany; patrick.scheiermann@med.uni-muenchen.de (P.S.); chefarztsekretariat@chkmb.de (D.B.); othmar.kofler@gmail.com (O.K.); klaus.hofmann-kiefer@med.uni-muenchen.de (K.H.-K.); 2 Department of Cardiac Surgery, University Hospital, LMU Munich, 81377 Munich, Germany; sven.Peterss@med.uni-muenchen.de (S.P.); christian.hagl@med.uni-muenchen.de (C.H.); 3 Department of Psychiatry, University Hospital, LMU Munich, 80336 Munich, Germany; daniela.eser@med.uni-muenchen.de; 4 Department of Anaesthesiology and Intensive Care Medicine, University Medical Center, 18057 Rostock, Germany; johannes.ehler@med.uni-rostock.de; 5 Clinic for Anaesthesia, Surgical Intensive Care, Emergency Medicine and Pain Therapy, Klinikum Frankfurt Hoechst, 65929 Frankfurt/Main, Germany; daniel.chappell@med.uni-muenchen.de

**Keywords:** delirium, cardiac surgery, off-pump surgery, natriuretic peptide, biomarker

## Abstract

*Background and Objectives:* Delirium is a common and major complication subsequent to cardiac surgery. Despite scientific efforts, there are no parameters which reliably predict postoperative delirium. In delirium pathology, natriuretic peptides (NPs) interfere with the blood–brain barrier and thus promote delirium. Therefore, we aimed to assess whether NPs may predict postoperative delirium and long-term outcomes. *Materials and Methods:* To evaluate the predictive value of NPs for delirium we retrospectively analyzed data from a prospective, randomized study for serum levels of atrial natriuretic peptide (ANP) and the precursor of C-type natriuretic peptide (NT-proCNP) in patients undergoing coronary artery bypass grafting (CABG) with or without cardiopulmonary bypass (off-pump coronary bypass grafting; OPCAB). Delirium was assessed by a validated chart-based method. Long-term outcomes were assessed 10 years after surgery by a telephone interview. *Results:* The overall incidence of delirium in the total cohort was 48% regardless of the surgical approach (CABG vs. OPCAB). Serum ANP levels > 64.6 pg/mL predicted delirium with a sensitivity (95% confidence interval) of 100% (75.3–100) and specificity of 42.9% (17.7–71.1). Serum NT-proCNP levels >1.7 pg/mL predicted delirium with a sensitivity (95% confidence interval) of 92.3% (64.0–99.8) and specificity of 42.9% (17.7–71.1). Both NPs could not predict postoperative survival or long-term cognitive decline. *Conclusions:* We found a positive correlation between delirium and preoperative plasma levels of ANP and NT-proCNP. A well-powered and prospective study might identify NPs as biomarkers indicating the risk of delirium and postoperative cognitive decline in patients at risk for postoperative delirium.

## 1. Introduction

Delirium is a common neuropsychological complication following cardiac surgery [1]. However, the pathogenesis of delirium is still not fully understood. To date, an interplay of neuronal aging with neuroendocrine dysregulation and oxidative stress, culminating in neuroendocrine dysregulation, is thought to be responsible for delirium [2]. Subsequently, an activation of brain microglia by peripheral inflammatory cytokines triggers a neuroinflammatory process that may lead to post-operative neuro-cognitive dysfunction (NCD), a long-term decline in cognitive performance that occurs one year after surgery at the earliest [3].

Endothelial damage is suspected to be the most important pathomechanism in evolving delirium and NCD, and was shown to have a direct association with cognitive dysfunction [4]. Natriuretic peptides (NPs) like atrial natriuretic peptide (ANP) and C-type natriuretic peptide (CNP) induce the breakdown of the vascular endothelial barrier [5]. Their role in the relaxation of the peripheral vasculature endothelium and its effects on coronary artery bypass grafts is well-known [6]. Additionally, ANP was shown to worsen disruption of the blood–brain barrier and brain edema in cases of traumatic brain injury [7].

Recently, NPs were shown to be associated with septic encephalopathy [8]. Not surprisingly, increased endothelial permeability, which may result from increased levels of NPs, is associated with delirium [9]. High systemic levels of NPs induce a down-regulation of NP receptors in the brain, resulting in dysregulation of synaptic transmission and plasticity, promotion of neuroinflammation and disruption of the blood–brain barrier [10]. Thus, as a primary objective of this study, we hypothesize that preoperatively increased levels of ANP and CNP enhance the vulnerability of the brain to inflammatory stimuli and serve as biomarkers for delirium [10,11,12].

Cardiac surgery bears a high incidence of delirium. The use and duration of a cardiopulmonary bypass (CPB) are thought to be responsible for an increased incidence of delirium and the acceleration of subsequent cognitive decline [13,14]. Delirium is associated with poor outcomes after coronary artery surgery with CPB (CABG) [15]. While most of the procedures in cardiac surgery require CPB, coronary surgery may also be performed without CPB, by means of off-pump coronary bypass grafting (OPCAB). Inconclusive data showed possible protective effects of OPCAB surgery in relation to the patients’ cognitive status [16]. Accordingly, OPCAB surgery reduced neurologic and clinical morbidity [17]. Still, little is known about favorable long-term cognitive outcomes after OPCAB surgery. As a secondary objective, we aimed to observe long-term cognitive outcomes in our cohort.

In order to evaluate the predictive capability of NPs for delirium, we first assessed ANP and N-terminal C-type natriuretic propeptide (NT-proCNP) serum concentrations before coronary artery surgery. Second, we assessed long-term outcomes of patients with delirium after coronary artery surgery in a pilot study.

## 2. Materials and Methods

This exploratory analysis of the previously-conducted prospective study was approved by the Institutional Ethics Board for the randomized controlled trial (trial registration no. 226-09, approved on 5 November 2010), and subsequently for the evaluation of the cognitive status and retrospective analysis of delirium (trial registration no. 62-16; medical faculty ethics committee, LMU Munich, Munich, Germany; approved on 5 February 2016). Written informed consent was obtained from each patient.

Thirty patients scheduled for elective coronary artery surgery were randomized to either a CABG or an OPCAB procedure. Of those, one patient was excluded due to failed evaluation of NP serum concentrations (due to insufficient plasma sample volume). One patient had an incomplete study chart and one patient (aged 18 years) was excluded because his age was out of a range of two standard deviations. Thus, 27 patients were included in the study cohort and evaluated with respect to the occurrence of postoperative delirium, plasma NP concentration and long-term cognitive outcomes (NCD). To factor out the potential harmful effects of CPB, we placed patients scheduled for elective coronary artery surgery in subgroups “CABG” and “OPCAB” and analyzed each group separately.

Surgical procedures and further anesthesia management were described in detail previously [18]. Briefly, all patients received oral premedication with 7.5 mg midazolam. Standardized intravenous induction of anesthesia consisted of midazolam, sufentanil, etomidate and rocuronium. Anesthesia was maintained with sufentanil and isoflurane (end-expiratory concentration 1.2 vol %). During CPB, ventilation was reduced to minimal ventilation with 100% oxygen. During OPCAB surgery, normal ventilation was maintained with 100% oxygen. Patients were transfused at the discretion of the anesthetist in charge and in accordance with our institutional procedures regarding blood transfusions in cardiac surgery patients [19]. For perioperative anticoagulation, heparin was used at the discretion of the surgeon in charge during CABG and OPCAB surgery, along with protamine sulfate to antagonize its effect at the end of the procedure. After surgery, patients were transferred to the intensive care unit and received piritramide for analgesia and a maximum of 2 mg/kg body weight of propofol per hour if a hypnotic was needed until the patient fulfilled common extubation criteria. No further midazolam was used for sedation.

For the analysis of NPs, we preoperatively measured plasma values of ANP and NT-proCNP under hemodynamically stable conditions after the induction of anesthesia.

Preoperative values of natriuretic peptides were determined as described previously [18]. For the detection of NT-proCNP, we used a sandwich enzyme-linked immunosorbent assay (Biomedica, Vienna, Austria) with a detection limit of 0.2 pmol/L. Patients’ blood samples were treated according to the manufacturer’s instructions. Although CNP is a paracrine and autocrine protein and is the most active NP in the human brain [20], NT-proCNP is a more stable precursor of CNP, which is predominant in the brain and excreted in equimolar amounts. NT-proCNP exactly reflects the total amount of CNP produced, whereas CNP is degraded quickly [21].

For the evaluation of delirium, we used the validated methods introduced by Inouye et al. [22] and Kuhn et al. [23]. The patients’ records were anonymized and 27 case vignettes—accurately made on the basis of medical and nursing commentaries, as well as the hospital discharge summary, hospital diagnoses and prescribed medication—were prepared by two independent chart abstractors. Both chart abstractors searched independently for evidence of acute mental confusion (e.g., delirium, mental status change, inattention, disorientation, hallucinations, agitation, inappropriate behavior, etc.) [21].

The abstracted charts were then presented at a consensus conference according to Kuhn et al. [23] and reviewed by five blinded independent delirium specialists (D.E.-V., O.K., N.B., P.S., C.N.) of different specialties (psychiatrist, cardiac anesthesiologist, registered intensive care nurse, intensive care physician, internal medicine intensivist). For every day in hospital, every expert stated either “no”, “possible delirium” or “probable delirium” for each individual case. A unanimous decision among the five reviewers was mandatory for the diagnosis of delirium. According to the individual diagnosis, a predominant delirium subtype was recorded. A patient was considered delirious if there was a consensus of “probable delirium” on each day.

To evaluate the long-term outcomes and evaluate for possible NCD, we performed a personal interview on the phone 10 years after surgery using the World Health Organization Disability Assessment Schedule 2.0 (WHODAS) [24] and the telephone version of the Short Blessed Test (SBT) [25]. WHODAS covers six domains (cognition, mobility, self-care, getting along, life activities, participation) converted into an overall functioning score ranging from 0 to 100 (0 = no disability, 100 = full disability). The SBT is a weighted six-item instrument which evaluates orientation, registration and attention, with a total score of 4 or less indicating normal cognition, between 5 and 9 indicating questionable impairment and 10 or more indicating cognitive impairment consistent with dementia. Pain was assessed as mean level of pain in the past two weeks (numeric rating scale with 10 indicating maximum pain). If the patient had passed away, the date of death was recorded.

### Statistical Analysis

Prior to the study, we calculated a sample size of 28 patients to be sufficient to show a difference in means in the concentration of ANP, given an effect size of 1 and an α-error of 5% with a power of 0.8 (Mann–Whitney U test, G*Power, University of Dusseldorf, Germany). To test for normal distribution of the data, the Kolmogorov–Smirnov Test was used. We present normally distributed data as mean ± standard deviation. Statistical differences were computed by a Student’s *t*-test. For non-normally distributed data we present median values (with 25th and 75th percentile) and used the Mann–Whitney U test. If adequate, we present 95% confidence intervals (CI). Categorical data were evaluated by a Chi-squared test and Fisher’s exact test, if necessary. For correlations, Spearman’s R was indicated. To detect the diagnostic specifics of NPs, we drew a receiver–operator curve (ROC) and specified the cut-off by Youden’s J. Mean differences were indicated as standard error (SEM) and with the corresponding CI. An α-error of 5% was supposed to be the border of significance (*p* < 0.05). For statistical analysis, SPPS Version 25.0 for Macintosh (IBM Corp., Armonk, NY, USA) was used.

## 3. Results

The demographic characteristics of the participants are shown in Table 1. We preoperatively detected a NT-proCNP level above 1.7 pg/mL in 12 of 13 postoperative delirious patients (see Table 2). With this threshold, NT-proCNP as a possible biomarker for delirium showed a 92.3% sensitivity (CI 64.0–99.8) and a 42.9% specificity (CI 17.7–71.1) with an area under the ROC of 0.643. With an ANP concentration of 64.6 pg/mL as a cut-off, we correctly identified all 13 delirious patients with 100% sensitivity (CI 75.3–100) and 42.9% specificity (CI 17.7–71.1). The concentration of NT-proCNP also correlates with longer delirium (0.405; *p* = 0.036). Cumulatively, patients with delirium received more packed red blood cells (2 versus 1 unit; *p* = 0.009). Comprising autologous blood re-transfusion, transfusion of fresh frozen plasma and thrombocyte concentrates (each not significant, *p* > 0.05), patients with delirium had a fluid balance twice as positive as non-delirious patients (*p* = 0.038). High preoperative ANP serum levels were positively correlated with the fluid balance (r = 0.471; *p* = 0.013).

Patients with delirium had received blood (n = 13/13) as often as patients without delirium (n = 10/14), a difference that was not statistically significant (*p* = 0.098). Significant differences reflecting the need for renal replacement therapy could not be detected in either preoperative or postoperative kidney function (as detected by serum concentration of creatinine) (Table 2).

Evaluating the long-term effects of delirium (NCD), 3 out of 13 (23%) formerly delirious patients and 6 patients out of 14 (43%) without delirium were successfully followed. There were no significant differences in pain or overall functioning as measured with the WHODAS 2.0 score (*p* = 0.231, Table 3). We observed SBT scores of 10 or more in patients that had been delirious, indicating dementia. Patients without delirium had lower SBT values, indicating better cognitive outcomes (*p* = 0.257).

Intraoperative and postoperative concentrations of NT-proCNP were positively correlated in both surgical methods (CPB 0.990; OPCAB 0.972; both *p* < 0.0001). In contrast to OPCAB surgery, NT-proCNP during CABG surgery increased significantly by 0.6 pmol/L (SEM 0.27; CI −1.158–−0.002; *p* = 0.049). No difference was observed in the incidence of postoperative delirium (CABG n = 6 (40%), OPCAB n = 7 (58%), *p* = 0.449), the length of stay in an intensive care unit, the in-hospital stay or long-term outcomes between CABG and OPCAB (see Supplementary Materials Tables S1 and S2).

## 4. Discussion

The results of the current study suggest that preoperatively elevated concentrations of ANP and NT-proCNP may be positive predictors of postoperative delirium in patients undergoing cardiac surgery regardless of the use of CPB (CABG vs. OPCAB). By design, we eliminated the impact of surgery and the use of CPB and found no difference between the different surgical strategies. For the evolution of delirium following cardiac surgery, delirium-inducing factors like surgical trauma, micro-emboli, hyperoxia and ischemia-reperfusion injury seem to be more important than the surgical approach (OPCAB or CPB). These individual factors are hard to grasp, which makes it worthwhile to examine potential biomarkers to assess individual risk. According to current delirium research, these individual, patient-specific characteristics are considered precipitants for delirium (amongst others), inducing a leakage of the blood–brain barrier (BBB) and eliciting a central neuroinflammatory response [2,26]. Failure to restore the integrity of the BBB in a timely fashion may trigger a long-term breakdown, an essential feature of subsequent cognitive decline. CNP and its predominator, NT-proCNP, serve as biomarkers for the integrity of the BBB [11]. It is known that systemic inflammation alters endothelial function, including the cerebral vasculature, allowing for the development of endothelial dysfunction. Validated biomarkers targeting endothelial dysfunction indicate vascular changes in sepsis and BBB impairment. C-type natriuretic peptide is dominantly expressed in the CNS and released during encephalopathy from endothelial cells [8]. Thus, its N-terminal propeptide (NT-proCNP) was recently validated as predictor of SAE, a pathologic situation comparable to delirium [26].

Most notably, all patients with postoperative delirium had increased preoperative concentrations of NPs. NPs increase endothelial permeability and consequently allow cytokines of peripheral (sterile or septic) inflammation to transmit into the brain, inducing neuronal inflammation followed by delirium [5,12,18]. Consequently, a preoperatively increased NP serum level might possibly serve as a predictor of postoperative delirium, as it reflects the vulnerability of the BBB. Elevated plasma markers of endothelial activation and BBB or neurological injury like NT-proCNP are associated with delirium as well [27]. Their high sensitivity documents the ability of NPs to serve as a predictor of delirium. However, specificity is low. Because a screening test should be optimized to detect as much potential subjects at risk as possible, the low specificity we found is acceptable for a non-invasive and effective test. As NP serum levels are easy to obtain and to quantify, future prospective studies should assess NPs before and during the course of cardiac surgery, together with a meticulous assessment of delirium and preoperative and postoperative deficits in neurocognition. We are to perform such a prospective study (DRKS00011833).

One could argue that in cardiac surgery CPB is accountable for increased delirium and increasing NT-proCNP, although recent studies showed no benefit of OPCAB in respect to the incidence of delirium [28]. Our findings also do no support this association. First, a highly positive fluid balance, often associated with CPB, was found to be associated with an increased incidence of postoperative delirium [29]. Second, as delirium incidence did not differ between OPCAB and CABG, it is unlikely that the perioperative increase of NT-proCNP in CPB promoted delirium. Moreover, the individual patients were prone to delirium and the surgical trauma (whether performed under CPB or OPCAB) induced delirium. Third, recent studies showed comparable incidences of delirium and severe postoperative complications for conventional OPCAB in contrast to CABG [28,30]. Fourth, with our design we eliminated the surgical aspect from the equation.

The prognostic value of transfusion on the evolution of delirium in patients has been studied before [31]. Regarding an association between transfusion and natriuretic peptides, it is well known that cardiac natriuretic peptides rise with volume overload. As we showed only preoperative values, perioperative transfusion could not have affected the values of natriuretic peptides prior to surgery. Additionally, there was no statistical difference in pre- or postoperative hemoglobin values.

As reported before, delirium has negative effects concerning short-term mortality and length of stay as well as short-term cognitive decline. For methodological reasons, the majority of studies do not proceed beyond the perioperative period, and a very small minority of studies report follow-up periods longer than five years. Despite the fact that our study’s results relating to long-term outcomes were not statistically significant (as this small pilot study only included and followed up a limited number of patients), we were able to present conclusive data on long-term outcomes which might promote further research on long-term outcomes after cardiac surgery. More patients with preoperatively elevated NT-proCNP died in the 10 years before follow-up. Again, comparable data showing NT-proCNP to increase mortality were observed in septic patients [7,32]. Altogether, future research should evaluate NT-proCNP as a prognostic biomarker for short- and long-term outcomes in cardiac surgery.

One possible limitation of the study is the small sample size and the retrospective assessment of delirium [7]. Nevertheless, with a well-validated chart-based method, we were able to adequately compensate for the lack of delirium screening in the retrospective analysis of the prospective study. However, retrospective evaluation methods for delirium are well established for the detection of delirium [22] and neuropsychological evaluation [33,34] and are helpful even compared with prospective methods [23]. Moreover, a retrospective setting always bears a potential bias and its results must be interpreted with care. However, for this pilot study, the retrospective approach appears to be valid.

## 5. Conclusions

In this prospective pilot study, we showed an association between NPs and delirium following cardiac surgery with and without CPB. Our hypothesis of NPs being potential biomarkers for delirium is worth pursuing in prospective clinical trials.

## Figures and Tables

**Table 1:** Characteristics of the study participants.

	No Delirium N = 14	Delirium N = 13	*p*-Value
sex, n (% male)	12 (85.7)	10 (76.9)	0.648
Age, years	64.3 ± 8.2	69.2 ± 4.6	0.067
Body mass index, kg/m^2^	25.67 ± 3.73	25.21 ± 2.92	0.725
Hemoglobin preoperative, g/dL	13.2 ± 1.9	12.9 ± 1.4	0.651
Preoperative reduced left ventricular function, n (%)	1 (7.1)	1 (7.7)	1.0
Preoperative creatinine, mg/dL	1.13 ± 0.30	1.18 ± 1.47	0.092

The table shows preoperative and basic perioperative parameters of the study participants (mean ± standard deviation or median (25th–75th percentile), respectively).

**Table 2:** Outcome variables.

	No Delirium N = 14	Delirium N = 13	*p*-Value
Preoperative ANP, pg/mL	71.11 ± 30.8	129.08 ± 79.6	0.003
Preoperative NT-proCNP, pmol/L	1.96 ± 0.88	4.47 ± 5.33	0.118
Duration of anesthesia, min	241 ± 27	275 ± 39	0.770
Bypass time, min	84 ± 57	109 ± 24	0.076
Artificial ventilation, h	18 (15–23)	22 (16–25)	0.302
Days in ICU	3 (2–5)	6 (4–8)	0.043
LoS, days	12 (10–19)	22 (17–28)	0.003
Crystalloids, mL	1250 (875–2000)	1500 (1000–2500)	0.325
Colloids, mL	500 (500–2000)	1000 (500–1500)	0.981
Transfusion (units) of			
- Intraoperative RBC	1 (0–2)	2 (2–3)	0.009
- Postoperative RBC	0 (0–1)	0 (0–1)	0.685
- FFP	3 (0–6.75)	6 (3–9.5)	0.185
- PC	0 (0–0.2)	1 (0–2.4)	0.116
- Autologous blood re-transfusion, mL	88.5 (0–494)	450 (110–875)	0.185
Hemoglobin postoperative, g/dl	9.9 (1.7)	9.4 (1.1)	0.372
Intraoperative fluid balance, mL	1625 (767–3256)	3200 (2015–4510)	0.038

The table shows peri- and postoperative variables in regard to the occurrence of postoperative delirium (mean ± standard deviation or median (25th–75th percentile), respectively). CPB: cardiopulmonary bypass; ICU: Intensive Care Unit; LoS: length of stay in the hospital; ANP: atrial natriuretic peptide; NT-proCNP: N-terminal-pro C-Type natriuretic peptide; RBC: unit of packed red blood cells; FFP: unit of fresh frozen plasma; PC: unit of platelet concentrate.

**Table 3:** Long-time outcome variables.

	No Delirium N = 14	Delirium N = 13	*p*-Value
Alive at follow up, n (%)	6 (43)	3 (23)	0.420
Alive for 5 years, n (%)	8 (57)	5 (39)	0.449
Median survival, years	8 (2–10)	3 (1–9)	0.280
Short Blessed test score, Mean (SD) (range)	7.7 (5.3) (0–12)	11 (1.2) (10–12)	0.257
WHODAS 2.0 score; Mean (SD) (range)	39 (11) (25–56)	52 (21) (35–83)	0.231
Numeric rating scale, Mean during past 14 days (range)	1 (1–4) (0–5)	0 (0–1) (0–3)	0.137

The table shows the values observed in a telephone interview ten years after coronary artery surgery. WHODAS World Health Organization Disability Assessment Schedule 2.0 (higher values indicating increased neuro-cognitive dysfunction) shows preoperative and basic perioperative parameters of the study participants (mean +/− standard deviation or median (25th–75th percentile), respectively).

## Supplementary Materials

The following are available online at https://www.mdpi.com/1010-660X/56/6/258/s1, Table S1: Characteristics of the study participants, Table S2: Long-term outcome variables.
