# Supplementary material for: Natriuretic Peptides as a Prognostic Marker for Delirium in Cardiac Surgery—A Pilot Study
Source: Medicina (Kaunas). 2020 May 27;56(6):258. doi: 10.3390/medicina56060258 (PMC7353880; doi:10.3390/medicina56060258)
Supplement: Supplementary file 1 [file medicina-56-00258-s001.pdf]

Supplementary Table 1: Characteristics of the study participants.

	CABG N = 15	OPCAB N = 12	*p*-Value
Sex, n (% male)	13 (86.7)	9 (75.0)	0.628
Age, years	65.2 (±6.8)	68.6 (±7.2)	0.217
Body mass index, kg/m^2	25.23 (±3.94)	25.72 (±2.44)	0.713
Hemoglobin preoperative, g/dL	13.2 (11.5–14.3)	13.3 (11.9–14.0)	0.516
Preoperative reduced left ventricular ejection fraction, n (%)	1 (6.7)	1 (8.3)	0.926
duration of anesthesia, min	259 (217–299)	251 (214–297)	0.867
artificial ventilation, h	19 (14–22)	22 (17–32)	0.139
days in ICU	3 (2–5)	6 (4–8)	0.021
LoS, days	19 (9–26)	16 (12–23)	0.648
preoperative creatinine, mg/dL	1.0 (0.9–1.2)	1.15 (1.0–1.7)	0.126

The table shows preoperative and basic perioperative parameters of the study participants in regard to the surgical method (mean ± standard deviation or median (25th–75th percentile), respectively). CABG: coronary artery surgery with cardiopulmonary bypass, OPCAB: Off-Pump Coronary Artery Bypass Grafting, ICU: Intensive Care Unit, LoS: length of stay in the hospital.

Supplementary Table 2: Long-term outcome variables.

	CABG<br>N = 15	OPCAB<br>N = 12	*p*-Value
Alive at follow up, n (%)<br>(Alive for 5 years, n (%)9	6 (40)<br>(9 (60)9	3 (25)<br>(4 (33)9	0.683<br>0.252
Median survival, years (percentiles)<br>(range9	8 (2–10)<br>(0-99	4 (1–9)<br>(0-79	0.252
Short blessed test score,<br>Median (percentiles) (range9	10 (2–10)<br>(0–109	12 (12–12)<br>(12–129	0.476
WHODAS 2.0 score (mean) (range9	40 (11)<br>(25–569	51 (22)<br>(33–839	0.305
Numeric rating scale, mean during past 14 days, (percentiles) (range9	1 (1–2)<br>(0–39	0 (0–4)<br>(0–59	0.352

The table shows peri- and postoperative variables in regard to the surgical method (mean ± standard deviation or median (25th–75th percentile), respectively). CABG: coronary artery surgery with cardiopulmonary bypass, OPCAB: Off-Pump Coronary Artery Bypass Grafting WHODAS: World Health Organization Disability Assessment Schedule 2.0 (higher values indicating increased neuro-cognitive dysfunction).
